# Transforming Growth Factor Beta (TGF*β*) Is Produced by and Influences the Proliferative Response of *Xenopus laevis* Lymphocytes

**DOI:** 10.1155/1993/63626

**Published:** 1993

**Authors:** Laura Haynes, Nicholas Cohen

**Affiliations:** Department ofMicrobiology and Immunology University of Rochester Medical Center Rochester New York 14642 USA

**Keywords:** TGF*β*, *Xenopus*, immunity, lymphocyte, amphibian, cytokine

## Abstract

Both TGF/β2 and 5 have been described in the South African clawed frog *Xenopus laevis* and have been cloned from the tadpole-derived fibroblast cell line, XTC. Because TGF*β* has such a profound inhibitory effect on the mammalian immune system, this study was performed to determine whether TGF*β*: (a) has any *in vitro* effects on the growth of *Xenopus* lymphoblasts, and (b) is produced by mitogen-activated *Xenopus* lymphocytes.

Following stimulation with mitogen or alloantigen, T lymphocytes from *Xenopus* secrete a T-cell growth factor (TCGF) that is functionally homologous to mammalian interleukin-2 (IL-2). Both recombinant human TGFβ1 and *Xenopus* TGF*β*5 inhibit TCGF-induced proliferation of *Xenopus* splenic blasts and this inhibition can be reversed with anti-pan TGF*β* antiserum. The Xenopus mitogen-induced saturated ammonium sulfate precipitated TCGF-containing supernatant (SAS TCGF SN) also contains latent TGF*β* as assayed on mink lung fibroblasts and *Xenopus* splenic blasts, and experiments utilizing anti-TGF*β* antiserum showed that only TGF*β*5 is present in this supernatant.


*Developmental Immunology*, 1993, Vol. 3, pp. 223–230
Reprints available directly from the publisher
Photocopying permitted by license only

© 1993 Harwood Academic Publishers GmbH
Printed in the United States of America

### Transforming Growth Factor Beta (TGF$\beta$) Is Produced by and Influences the Proliferative Response of *Xenopus laevis* Lymphocytes

LAURA HAYNES and NICHOLAS COHEN*

*Department of Microbiology and Immunology, University of Rochester Medical Center, Rochester, New York 14642*

Both TGF$\beta$2 and 5 have been described in the South African clawed frog *Xenopus laevis* and have been cloned from the tadpole-derived fibroblast cell line, XTC. Because TGF$\beta$ has such a profound inhibitory effect on the mammalian immune system, this study was performed to determine whether TGF$\beta$: (a) has any *in vitro* effects on the growth of *Xenopus* lymphoblasts, and (b) is produced by mitogen-activated *Xenopus* lymphocytes.

Following stimulation with mitogen or alloantigen, T lymphocytes from *Xenopus* secrete a T-cell growth factor (TCGF) that is functionally homologous to mammalian interleukin-2 (IL-2). Both recombinant human TGF$\beta$1 and *Xenopus* TGF$\beta$5 inhibit TCGF-induced proliferation of *Xenopus* splenic blasts and this inhibition can be reversed with anti-pan TGF$\beta$ antiserum. The *Xenopus* mitogen-induced saturated ammonium sulfate precipitated TCGF-containing supernatant (SAS TCGF SN) also contains latent TGF$\beta$ as assayed on mink lung fibroblasts and *Xenopus* splenic blasts, and experiments utilizing anti-TGF$\beta$ antiserum showed that only TGF$\beta$5 is present in this supernatant.

KEYWORDS: TGF$\beta$, *Xenopus*, immunity, lymphocyte, amphibian, cytokine.

### INTRODUCTION

TGF$\beta$ is a pleiotrophic cytokine produced by a number of different cell types including platelets, macrophages, fibroblasts, and T and B lymphocytes. TGF$\beta$ is secreted in a 100-kD biologically inactive latent form. This latency peptide can be cleaved by a change in pH or proteolysis to yield a 25-kD disulfide-bonded homodimer (reviewed in Roberts and Sporn, 1990). Five types of TGF$\beta$ have been cloned: TGF$\beta$1, 2, and 3 were originally described in humans (Assoian et al., 1983; Wrann et al., 1987; ten Dikje et al., 1988); TGF$\beta$4 has been found only in chickens (Jakowlew et al., 1988); TGF$\beta$5 has been found only in *Xenopus* (Roberts and Sporn, 1990). The amino-acid-sequence identity of the aforementioned processed TGF$\beta$s (after cleavage of latency peptide) is between 60% and 80%. TGF$\beta$5 is 76% identical to TGF$\beta$1, 66% identical to TGF$\beta$2, 69% identical to TGF$\beta$3, and 72% identical to TGF$\beta$4. Regions of identity include a highly conserved site and nine conserved cleavage cysteine residues. TGF$\beta$ 1 through 5 also show functional conservation in a number of assays, including the inhibition of proliferation of mink lung fibroblasts (MLF) and the stimulation of normal rat kidney (NRK) fibroblast colony formation in the presence of epidermal growth factor (Roberts and Sporn, 1990).

TGF$\beta$ plays a role in the control of the formation of extracellular matrices, myogenesis, formation and remodeling of bone, and in embryogenesis. TGF$\beta$ also functions in the immune system. It inhibits T- and B-cell proliferation, NK-cell activity, and generation of mixed lymphocyte responses and cytotoxic T cells (Massague, 1990). Kehrl et al. (1986) reported that TGF$\beta$ is secreted and TGF$\beta$ receptors are expressed by mitogen-activated T cells. These and other investigators believe that TGF$\beta$ acts to limit T- and B-cell clonal expression and to stimulate fibroblast proliferation to regulate inflammation and promote healing.

TGF$\beta$2 and 5 have been cloned from the *Xenopus* tadpole-derived fibroblast cell line, XTC,

*Corresponding author.

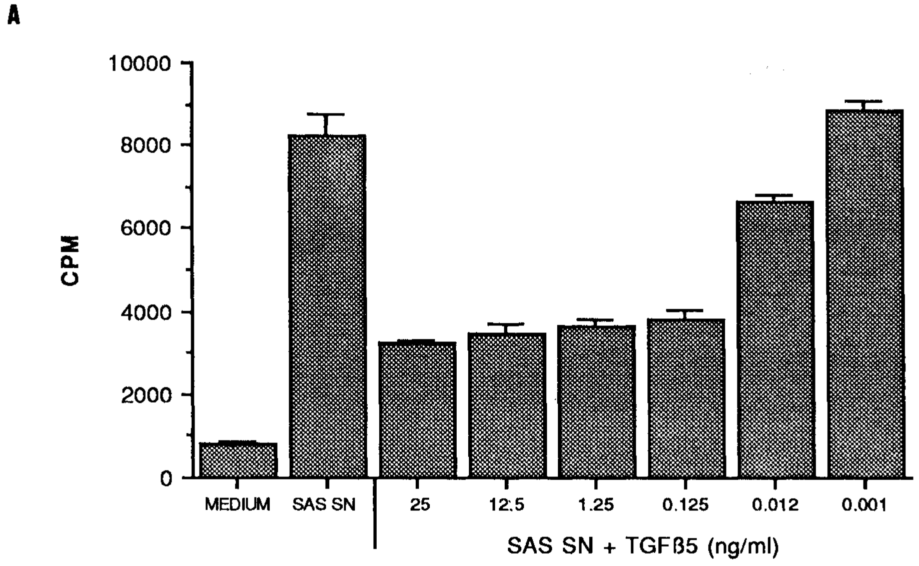

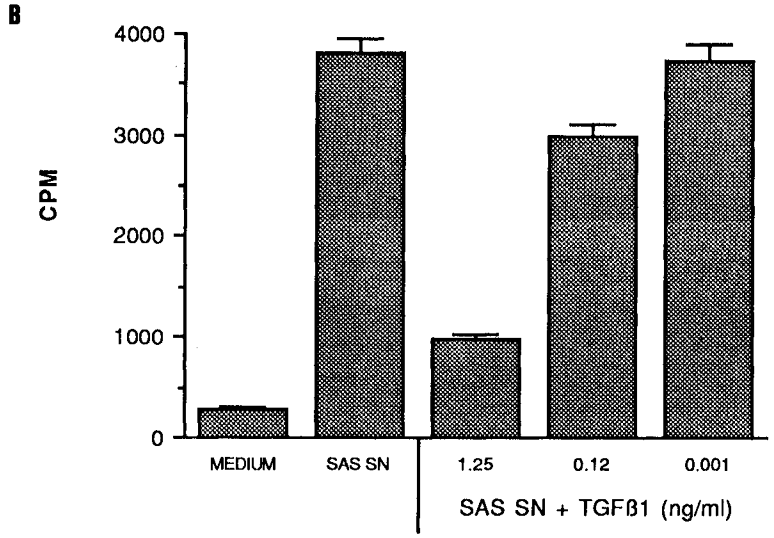

FIGURE 1. TGF$\beta$ inhibits TCGF-induced proliferation of splenic blasts. Representative experiment showing 3-day-old *Xenopus* splenic blasts assayed with 25% SAS TCGF SN alone or with the indicated concentrations of *Xenopus* rTGF$\beta$5 (**A**) or human rTGF$\beta$1 (**B**) in a 3-day $^3$H-thymidine incorporation assay. The results are expressed as mean CPM± SE.

and they have mesoderm-inducing activity on explants of amphibian ectoderm (Roberts and Sporn, 1990). Because TGF$\beta$ has such a profound effect on the mammalian immune system, this study was performed to determine whether TGF$\beta$ has any *in vitro* effects on the growth of *Xenopus* lymphoblasts and if it is produced by mitogen-activated *Xenopus* lymphocytes.

### RESULTS

*Xenopus* SAS TCGF SN contains a TCGF that is functionally homologous to mammalian interleukin-2 (IL-2) (Watkins and Cohen, 1987; Haynes and Cohen, 1993). One of its main *in vitro* activities is the induction of proliferation of activated, but not resting, splenocytes. Recombinant *Xenopus* TGF$\beta$5 (rTGF$\beta$5) inhibited the SAS TCGF SN-induced proliferation of 3-day-old *Xenopus* splenic blasts (Fig. 1A). Maximum inhibition was seen with 0.125 ng/ml and this inhibition could be titrated until no inhibition was seen with 0.001 ng/ml. Similar results were obtained with recombinant human TGF$\beta$1 (Fig. 1B).

To determine whether the inhibition of lymphoblast proliferation associated with rTGF$\beta$5 resulted from TGF$\beta$5 or from some contaminant

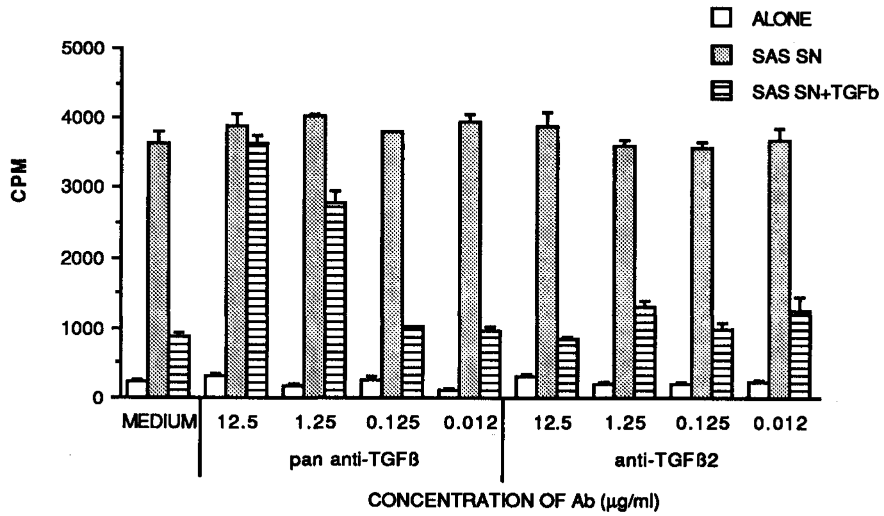

FIGURE 2. Anti-pan TGF$\beta$ antiserum reverses TGF$\beta$-induced inhibition of TCGF-induced proliferation. Three-day-old *Xenopus* splenic blasts were cultured alone or with 25% SAS TCGF SN and 1.25 ng/ml rTGF$\beta$5 with or without the indicated concentrations of rabbit anti-pan TGF$\beta$ or rabbit anti-TGF$\beta$2 antiserum, as described in Materials and Methods, in a 3-day $^{3}$H-thymidine incorporation assay. Results are expressed as mean CPM ±SE.

in the TGF$\beta$5 preparation, TGF$\beta$5 was preincubated with anti-pan TGF$\beta$ antiserum and then assayed (Fig. 2). Whereas the anti-pan TGF$\beta$ antiserum reversed the inhibition of proliferation, the control anti-TGF$\beta$2 antiserum (which does not recognize TGF$\beta$5) did not. The observed inhibition of proliferation, therefore, was specifically due to TGF$\beta$5. Neither antibody was mitogenic for *Xenopus* blasts (Fig. 2, open bars).

The next set of experiments tested for the presence of TGF$\beta$ in the SAS TCGF SN by assaying acid-treated (to activate latent TGF$\beta$) SAS TCGF SN for biological activity on mink lung fibroblasts and *Xenopus* splenic blasts. Figure 3A demonstrates that rTGF$\beta$5 inhibited the proliferation of MLF in a dose-dependent manner; Fig. 3B reveals that the SAS TCGF SN exhibited similar inhibitory activity, but only upon acid activation. Thus, PHA-stimulated *Xenopus* splenocytes secrete both TCGF and a latent form of TGF$\beta$. Indeed, the undiluted SAS TCGF SN exhibits activity equivalent to 125 pg/ml of rTGF$\beta$5.

Figure 4 shows that acid-activated SAS TCGF SN also induced reduced proliferation of *Xenopus* splenic blasts when compared to untreated SN. This reduction resulted from TGF$\beta$ activity and not from denaturation of the TCGF molecule during the acid treatment, because the full stimulatory activity of the SAS TCGF SN could be restored when it was first treated with the anti-pan TGF$\beta$ antiserum (Fig. 5).

Finally, because both TGF$\beta$2 and 5 have been described in *Xenopus* (Roberts and Sporn, 1990), antiserum specific for each of these proteins was used in an attempt to neutralize the TGF$\beta$ biological activity found in the acid-treated SAS TGCF SN. Figure 6 shows that the anti-TGF$\beta$2 has no effect, whereas the anti-TGF$\beta$5 reverses the inhibitory activity found in the acid-treated supernatant.

### DISCUSSION

In mammals, TGF$\beta$ is produced by mitogen-activated T cells, inhibits IL-2-dependent T-cell proliferation, and downregulates the immune response (Kehrl et al., 1986). Our study shows that these observations also are applicable to the *Xenopus* immune system. *Xenopus* splenic blasts proliferate in response to TCGF(s) present in mitogen-induced SAS TCGF SN and this proliferation can be inhibited by either rTGF$\beta$ (1 or 5) or by activating the latent TGF$\beta$ that is present in the supernatant. In blocking experiments with an anti-pan TGF$\beta$ antiserum, this inhibition was shown to result from TGF$\beta$. Results of an additional two experiments with antisera specific for either TGF$\beta$2 or TGF$\beta$5 are consistent with the proposition that the inhibitory effects of the acid-activated TCGF-rich supernatant are due solely to TGF$\beta$5. That is, there was no reversal of inhibition with anti-TGF$\beta$2; the extents to which the anti-pan TGF$\beta$ antiserum and the anti-TGF$\beta$ 5-specific antiserum reversed inhibition were similar, and the lymphoblast growth-promoting activity of the acid-activated supernatant was completely restored with the anti-TGF$\beta$5-specific

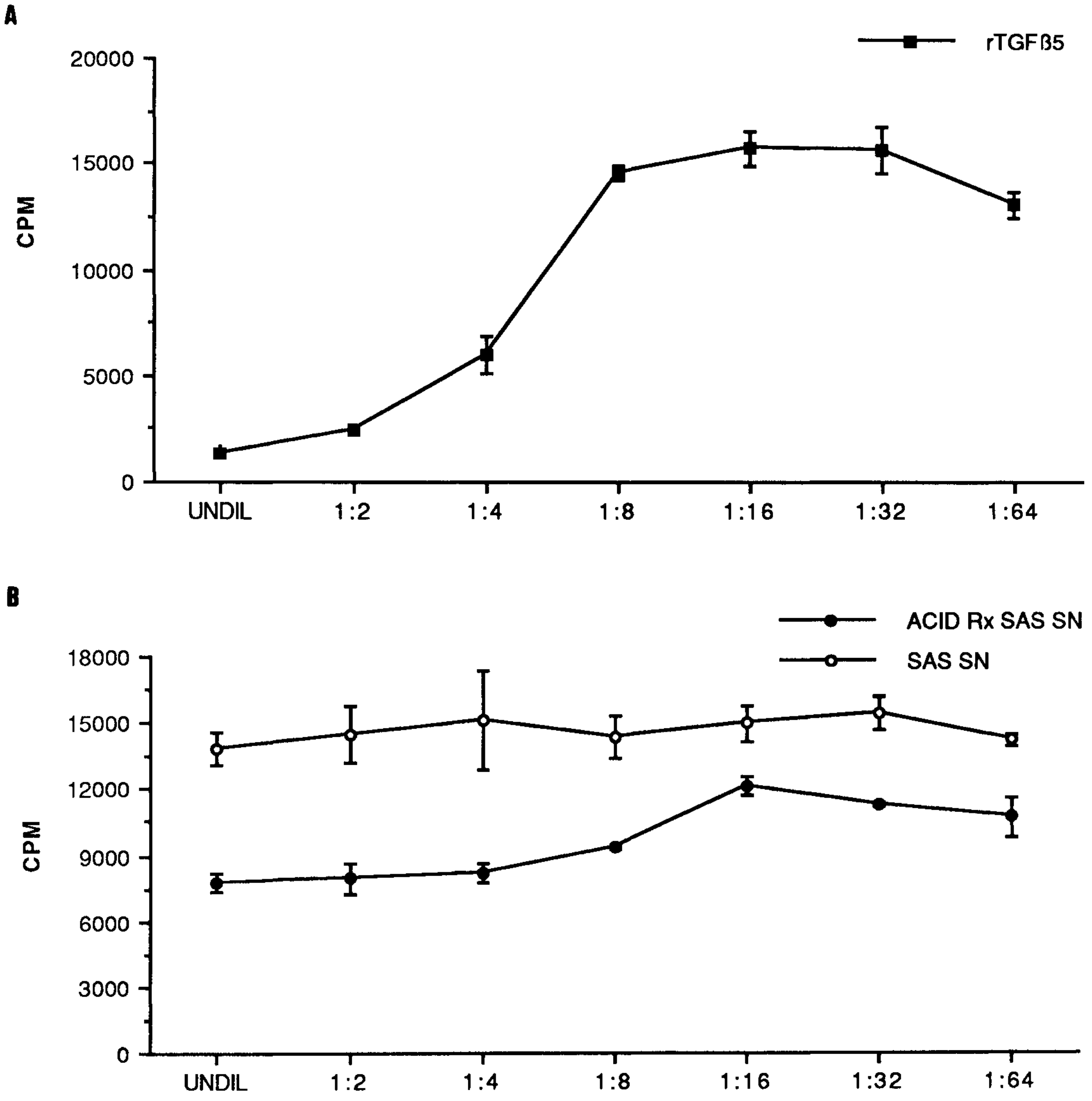

FIGURE 3. The effect of *Xenopus* TGF$\beta$ on mink lung fibroblasts. *Xenopus* rTGF$\beta$5 (**A**) (starting concentration=0.5 ng/ml) and *Xenopus* SAS TCGF SN, untreated or acid treated (acid Rx) (**B**) were assayed on cultures of MLF, as detailed in Materials and Methods, for 24 hr and the cultures were then pulsed with $^3$H-thymidine for 6 hr. Results are expressed as mean CPM±SE. For both experiments, CPM with medium alone was 9013.8±775; acid treatment of medium has no effect.

antiserum treatment. Whether "all" supernatants prepared from mitogen- or antigen-stimulated larval as well as adult *Xenopus* splenocytes also contain only TGF$\beta$5 is currently being investigated. Because TGF$\beta$ is involved in development, we are also investigating its role in the immune system during metamorphosis to determine if the downregulation of the immune response that is seen during this period (Flajnik et al., 1987) is, at least in part, attributable to TGF$\beta$.

TGF$\beta$ activity in the SAS TCGF SN was demonstrated by the ability of an acid-activated, but not an untreated, supernatant to inhibit the proliferation of mink lung fibroblasts. It is not surprising to find that both human rTGF$\beta$1 and frog rTGF$\beta$5 exhibit biological activity on frog and mammal cells because the three types of TGF$\beta$ receptors (I, II, and III) bind all types of TGF$\beta$, albeit with different affinities (Cheifetz et al., 1988; Roberts and Sporn, 1990).

Like mammals, *Xenopus* has T and B lymphocytes and expresses class I and class II MHC molecules that function much like mammalian MHC glycoproteins (Du Pasquier, 1989; Flajnik and Du

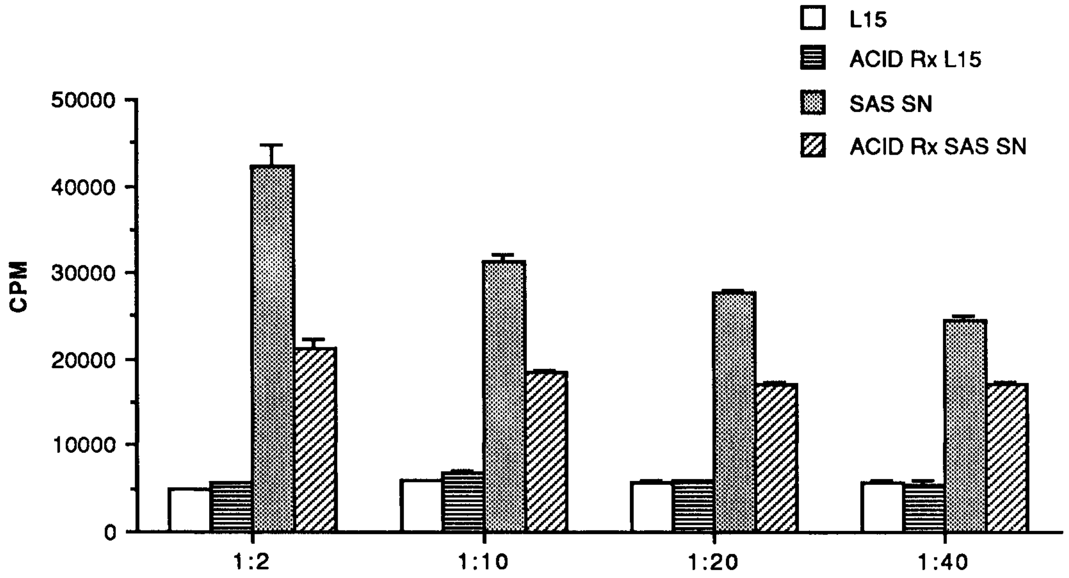

FIGURE 4. SAS TCGF SN contains TGF$\beta$ inhibitory activity for *Xenopus* splenic blasts. Untreated or acid Rx ASA TCGF SN, at the indicated concentrations, was assayed on 3-day-old *Xenopus* splenic blasts in a 3-day $^{3}$H-thymidine incorporation assay. Results are expressed as mean CPM±SE. L15 is the complete frog medium with 0.25% BSA.

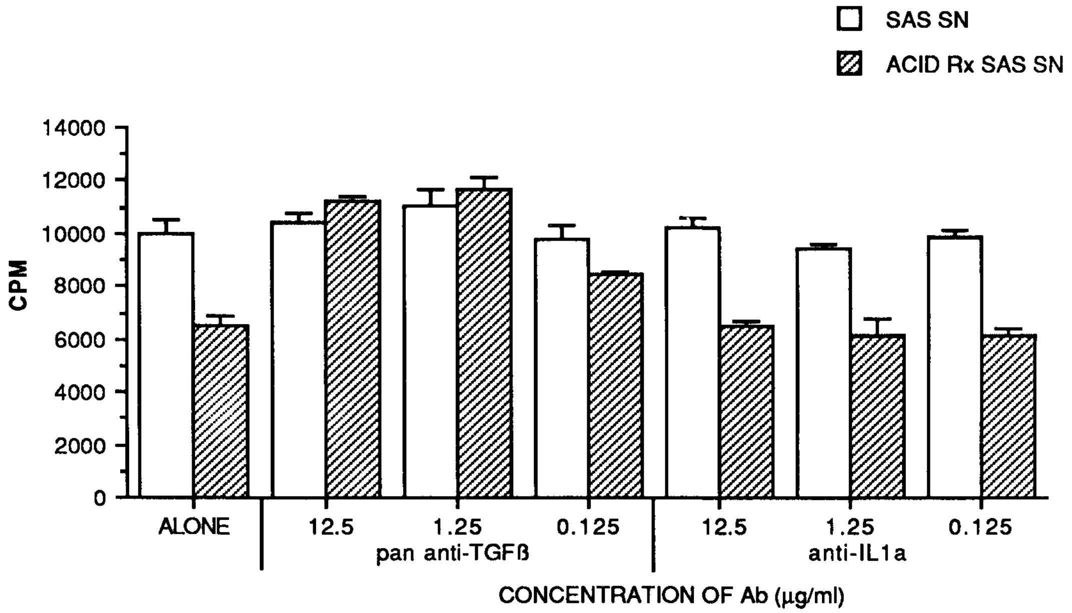

FIGURE 5. Anti-pan TGF$\beta$ antiserum reverses TGF$\beta$ inhibitory activity found in SAS TCGF SN. Untreated or acid Rx SAS SN (25% per well) with or without rabbit anti-pan TGF$\beta$ or control rabbit anti-IL-1a antiserum, at the indicated concentrations, was assayed (as described in Materials and Methods) on 3-day-old *Xenopus* splenic blasts in a 3-day $^{3}$H-thymidine incorporation assay. Results are expressed as mean CPM±SE.

Pasquier, 1990). *Xenopus* leukocytes produce an IL-1-like cytokine (Watkins et al., 1987). *Xenopus* T cells produce additional cytokines involved in T- and B-cell proliferation (Cohen and Haynes, 1991) and also exhibit MHC-restricted cytotoxicity (Harding, 1990). *Xenopus* B cells can secrete one of three types of immunoglobulin during an immune response, which are homologous with

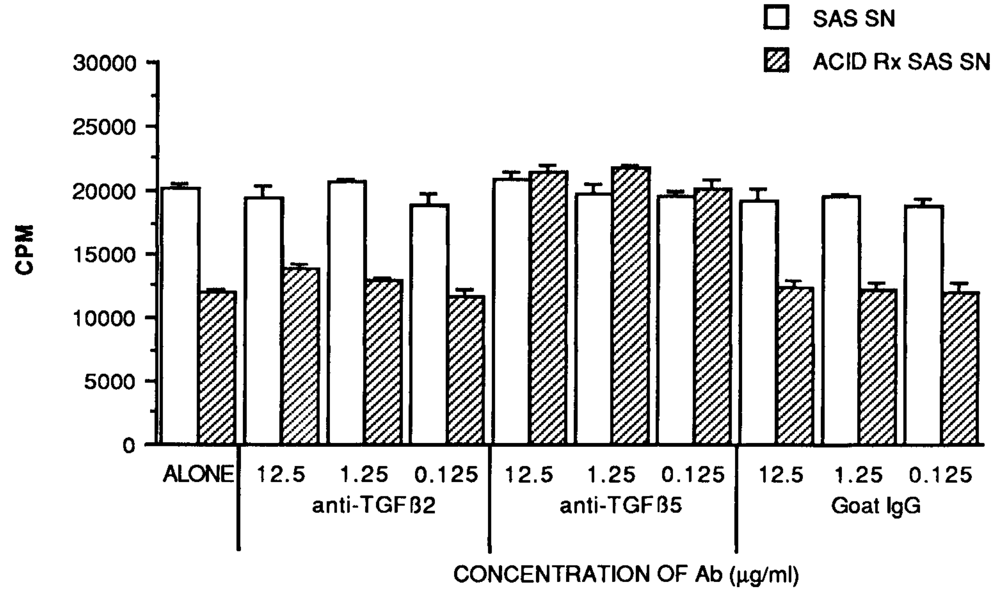

FIGURE 6. Anti-TGF$\beta$5, but not anti-TGF$\beta$2, antiserum reverses TGF$\beta$ inhibitory activity found in SAS TCGF SN. Untreated or acid Rx SAS SN (25% per well) with or without goat anti-TGF$\beta$2, goat anti-TGF$\beta$5, or control goat IgG, at the indicated concentrations, was assayed (as described in Materials and Methods) on 3-day-old *Xenopus* splenic blasts in a 3-day $^3$H-thymidine incorporation assay. Results are expressed as mean CPM±SE. CPM with medium alone is 2061.5±80.1.

mammalian IgM, IgG, and IgA (Du Pasquier, 1989). By demonstrating that *Xenopus* lymphocytes produce TGF$\beta$ that can downregulate lymphocyte proliferation, the present study provides additional evidence for the remarkable similarity of the *Xenopus* and mammalian immune systems.

### MATERIALS AND METHODS

### Animals

Fully grown adult female *Xenopus laevis* were purchased from Xenopus 1 (Ann Arbor, Michigan) or the South African Xenopus Facility (Noordhoek, South Africa).

### Production of SAS TCGF SN

SAS TCGF SN was generated as previously described (Watkins and Cohen, 1987). Briefly, 5x $10^6$ *Xenopus* splenocytes/ml were incubated at 26°C in complete medium [Leibovitz's L-15 medium (Gibco, Grand Island, New York) adjusted to amphibian osmolarity (220 mOsm) and supplemented with $1.25\times10^{-5}$ M HEPES buffer (Gibco), 100 U/ml penicillin, 100 $\mu$g/ml streptomycin (Gibco), $1\times10^{-2}$ M $NaHCO_3$, 5x $10^{-5}$ M 2-mercaptoethanol (Sigma, St. Louis, Missouri)] with 0.25% bovine serum albumin (BSA) and 1 $\mu$g/ml PHA-P conjugated to agarose beads (Sigma). Supernatants were collected from 24-hr and 48-hr cultures, pooled, and the PHA beads were removed from the supernatant by centrifugation. The resulting supernatant was precipitated with saturated ammonium sulfate, dialyzed (Spectra/Por membrane, $M_r$ cutoff 6000–8000) with APBS, and sterile filtered before use. All experiments described in this paper were performed with a single batch of SAS TCGF SN.

To activate any latent TGF$\beta$ present in the SAS TCGF SN, it was subjected to acid activation. Five hundred microliters of SN or complete medium was treated with 5 $\mu$l of 3N NCl for 1 hr at room temperature; the pH was neutralized with 5 $\mu$l of 3N NaOH. Samples were concentrated using Centricon Microconcentrators (Amicon, Beverly, Massachusetts) with a $M_r$ cutoff of 10,000 and then assayed.

### Preparation and Assay of Splenic Blasts

Splenocytes ($5\times10^6$/ml) were cultured in complete medium with 10% heat-inactivated fetal bovine serum (FBS) (Hyclone, Logan, Utah) and

1 to 2 μg/ml PHA-P (Sigma) for 3 days at 26°C in 24-well plates (Costar, Cambridge, Massachusetts). The resulting blasts were then centrifuged (350×g) over Histopaque $\delta$=1.077 (Sigma) and washed twice in complete medium with 1% FBS. TCGF activity was assayed on splenic blasts in a 3-day $^3$H-thymidine incorporation assay. The samples were incubated with 5× $10^4$ blasts per well (in complete L-15 with 1% FBS) in 96-well round-bottom plates (Costar). For antibody-inhibition experiments, samples were incubated with antibody for 1 hr in the well before the addition of the cells. The rabbit anti-porcine TGFβ2 polyclonal antiserum (R & D Systems, Minneapolis, Minnesota) specifically neutralizes biological activities of TGFβ2 and 3; rabbit anti-pan-TGFβ polyclonal antiserum (R & D Systems) neutralizes the biological activities of TGFβ1, 2, 3, and 5. Goat anti-purified porcine TGFβ2 and anti-recombinant *Xenopus* TGFβ5, also purchased from R & D Systems, were only used to identify the type of TGFβ in the SAS TCGF SN (see Fig. 6). Rabbit anti-human IL-1α (Genzyme, Boston, Massachusetts) and goat IgG (Sigma) were also used as a negative control. After 48 hr, 1 μCi/well $^3$H-thymidine (Amersham, Arlington Heights, Illinois) was added. The cultures were harvested after 72 hr and processed for liquid scintillation spectrometry. All cultures were plated in triplicate and the data are presented as mean counts per minute (CPM)±SE.

### Mink Lung Fibroblasts (MLF)

MLF (Mv1Lu CCL-64, originally from ATCC; a gift from the laboratory of Dr. David Scott) were maintained at 37°C in 25-cm$^2$ tissue-culture flasks (Costar) in complete mouse medium [RPMI-1640 (Gibco), 100 U/ml penicillin, 0.1 mg/ml streptomycin, 2 mM glutamine, 0.1 mM nonessential amino acids, 10 mM HEPES, 1 mM sodium pyruvate, $4.4\times10^{-2}$ M sodium bicarbonate, 0.04 mM 2-mercaptoethanol] with 5% FBS. To assay for TGFβ activity, fibroblasts were aliquoted ($2\times10^4$ per well) in complete medium with 5% FBS in 96-well flat-bottom plates (Costar) and allowed to become confluent. The medium was then replaced with serum-free complete mouse medium and the cells were incubated overnight. The serum-free medium was removed and TGFβ (recombinant human TGFβ1 from R & D Systems was provided by Dr. David Scott; recombinant *Xenopus* TGFβ5 was purchased from R & D Systems) or the supernatants to be tested were added to the cultures that were then incubated for 24 hr. Each well was then pulsed with 1 μCi $^3$H-thymidine (Amersham) for 6 hr and freeze/thawed before harvesting and processing for liquid scintillation spectrometry. All assays were plated in triplicate and the data are presented as the mean CPM±SE.

### ACKNOWLEDGMENTS

We greatly appreciate Dr. David Scott and Dr. Sally Kent of the University of Rochester for providing the mink lung fibroblasts and the rTGFβ1 and for guidance with the assays. We would also like to thank Anne Koniski for her skillful technical assistance.

*(Received January 27, 1993)*

*(Accepted March 11, 1993)*

### REFERENCES

Assoian R.K., Komoriya A., Meyers C.A., Miller D.M., and Sporn M.B. (1983). Transforming growth factor-beta in human platelets. J. Biol. Chem. **258:** 7155–7160.

Cheifetz S., Bassols A., Stanely K., Ohta M., Greenberger J., and Massague J. (1988). Heterodimeric transforming growth factor beta. Biological properties and interaction with three types of cell surface receptors. J. Biol. Chem. **263:** 10783–10789.

Cohen N., and Haynes L. (1991). The phylogenetic conservation of cytokines. *In:* Phylogenesis of immune function, Warr G.W., and Cohen N. Eds. (Boca Raton, FL: CRC Press), pp. 241–268.

Du Pasquier L. (1989). Evolution of the immune system. *In:* Fundamental immunology, 2d ed., Paul W.E. Ed. (New York: Raven Press), pp. 139–165.

Flajnik M.F., and Du Pasquier L. (1990). The major histocompatibility complex of frogs. Immunol. Rev. **113:** 45–63.

Flajnik M.F., Hsu E., Kaufman J.F., and Du Pasquier L. (1987). Changes in the immune system during metamorphosis of *Xenopus*. Immunol. Today **8:** 58–64.

Harding F.A. (1990). Molecular and cellular aspects of the immune system of lower vertebrates. Doctoral dissertation, University of Rochester, Rochester, New York.

Haynes L., and Cohen N. (1993). Further characterization of an interleukin-2-like cytokine produced by *Xenopus laevis* T lymphocytes. Devel. Immunol. (In press.)

Jakowlew S.B., Dillard P.J., Sporn, M.B., and Roberts A.B. (1988). Complementary deoxyribonucleotide acid cloning of an mRNA encoding transforming growth factor-beta 4 from chicken chondrocytes. Mol. Endocrinol. **2:** 1186–1195.

Kehrl J.H., Wakefield L.M., Roberts A.B., Jakowlew S., Alvarez-Mon M., Derynck R., Sporn M.B., and Fauci A.S. (1986). Production of transforming growth factor β by human T lymphocytes and its potential role in the regulation of T cell growth. J. Exp. Med. **163:** 1037–1050.

Massague J. (1990). The transforming growth factor-$\beta$ family. Ann. Rev. Cell Biol. **6:** 597–641.

Roberts A.B., and Sporn M.B. (1990). The transforming growth factor $\beta$s. *In:* Peptide growth factors and their receptors I. Sporn, M.B., and Roberts A.B. Eds. (New York: Springer-Verlag), pp. 417–472.

ten Dijke P., Hanson P., Iwata K.K., Pieler C., and Foulkes J.G. (1988). Identification of a new member of the transforming growth factor-$\beta$ gene family. Proc. Natl. Acad. Sci. USA **85:** 4715–4719.

Watkins D., and Cohen M. (1987). Mitogen-activated *Xenopus laevis* lymphocytes produce a T-cell growth factor. Immunology **62:** 119–125.

Watkins D., Parsons S.C., and Cohen N. (1987). A factor with interleukin-1-like activity is produced by peritoneal cells from the frog, *Xenopus laevis.* Immunology **62:** 669–673.

Wrann M., Bodmer S., de Martin R., Siepl C., Hofer-Warbinek R., Frei K., Hofer E., and Fontana A. (1987). T cell suppressor factor from human glioblastoma cells is a 12.5 KD protein closely related to transforming growth factor beta. EMBO J. **6:** 1633–1636.

